# Contamination and Health Risk Assessment of Heavy Metals in Soil and Ditch Sediments in Long-Term Mine Wastes Area

**DOI:** 10.3390/toxics10100607

**Published:** 2022-10-13

**Authors:** Bo Li, Jiangdi Deng, Zuran Li, Jianjun Chen, Fangdong Zhan, Yongmei He, Lu He, Yuan Li

**Affiliations:** 1 College of Resources and Environment, Yunnan Agricultural University, Kunming 650201, China; 2 Faculty of Animal Science and Technology, Yunnan Agricultural University, Kunming 650201, China; 3 College of Horticulture and Landscape, Yunnan Agriculture University, Kunming 650201, China

**Keywords:** mining wasteland, ditches, sediments, heavy metals, ecological risks, health risks

## Abstract

The ecological and health risks posed by wastes discharged from mining areas to the environment and human health has aroused concern. 114 soil samples were collected from nine areas of long-term mine waste land in northwestern Yunnan to assess the pollution characteristics, ecological and health risks of heavy metals. The result revealed that the geo-accumulation indexes were Cd (4.00) > Pb (3.18) > Zn (1.87) > Cu (0.25). Semi-variance analysis revealed that Cd and Cu showed moderate spatial dependency, whereas Pb and Zn showed strong spatial dependency. Cd posed an extreme potential ecological risk. Slopes and ditches were extreme potential ecological risk areas. Non-carcinogenic risk to children from Pb and Carcinogenic risk to adult and children from Cd was non-negligible and direct ingestion was the major source. This study provided a scientific basis for policymakers in management and exposure reduction.

## 1. Introduction

Heavy metal pollution is of wide concern worldwide, and the distribution of heavy metal pollution and ecological risk assessment has been paid increasing attention with nations, administrative regions, waters and road networks as the basic units, and rivers, lakes, mining areas, industrial areas and farmland as the main research subjects [1,2]. Multiple studies have shown that mining areas and industrial areas exhibited a higher geo-accumulation index and ecological risk index than other functional areas [3,4,5]. The sources of heavy metals in rivers and lakes are greatly heterogeneous due to different industrial structures and production methods. Mining and rock weathering were the main factors for heavy metal pollution in rivers and lakes in Asia [6]. Soil heavy metal pollution in mining areas in China showed a strong geographical distribution due to mining, smelting emissions and human activities and a higher geochemical background. Soil heavy metal pollution was mostly found in southern and eastern China and lead-zinc mine tailings were one of the main sources of pollution [7,8].

Heavy metal emissions in China have decreased since 2012. However, farmland around the mining and smelting areas accumulated a certain number of heavy metals, especially the continuous accumulation of Cd and Hg [9]. The characteristics of soil heavy metal accumulation around mining areas were influenced by topographic factors (elevation and slope), as well as natural factors (landscape, wind, rainfall and water flow). The heavy metals around the mine area were affected by wind dispersal to 2 km downwind. Heavy metal distribution was affected by water flow redistribution in rivers [10]. The distribution of Pb, Zn and Cu within 800 m downstream of the river was affected by water flow scouring, which caused some degree of landscape degradation [11,12]. The movement of As, Cd and Pb in soil was influenced by tillage behavior and transported to farmland 4 km away [13]. Mining waste areas posed a continuous threat to surrounding agricultural land and rivers. Anthropogenic activities and water flow scouring caused accumulation of heavy metals in soil at river confluences. The distribution of heavy metals at the river scale showed different accumulation characteristics for different elements. Cu, Fe and Mn accumulated in the middle of the riverbed and riverbanks, Pb mainly concentrated in the middle of the riverbed and Zn concentration was high at riverbanks [14]. The period of mine closure had a strong influence on the distribution of heavy metals in river watersheds. Heavy metal elements migrated to the surrounding environment as particles in a short period of time. Fine grained sediments were the main source of river pollutants. Cd continuously leached from contaminated valley bottoms and migrated to water areas 4 km away [15]. The extent of heavy metal pollution in mining areas was continuously influenced by natural factors and anthropogenic activities.

Direct ingestion, dermal contact and inhalation absorption were the main routes of human exposure to soil heavy metals [16]. Heavy metals were deposited in soils by atmospheric deposition, industrial emission and erosion and enter the food chain through contaminated crops and animals. They could also enter the human body through breathing and skin contact. Heavy metals (Cd, Pb, As, etc.) entering the human body destroyed protein activity, led to metabolic disorders in the human body and resulted in kidney damage, developmental disorders, cardiovascular disease, cancer and other diseases [17].

Pollution indexes were used for the evaluation of contamination levels. There were more than 18 commonly used indexes (*I_geo_*, *PI*, *EF*, *PINemerow*, *PLI*, *RI*, *mCd*, etc.), which were mainly divided into pairs of individual and integrated evaluation methods [18]. These evaluation methods were used in a wide range of scenarios and were suitable for soil contamination/ecological risk assessment. Researchers developed a methodology for integrated soil risk assessment in industrial and mining areas, generating a complete regional map of soil risk [19]. The assessment of the spatial distribution and contamination status of heavy metals in agricultural soil after irrigation with river water located downstream of the mining area showed that agricultural farming was the main source of pollutants. Hg was probably transported from the upstream gold mine by atmospheric conditions and rivers, and Ni by a combination of agricultural measures and mining [2]. These methods fully demonstrated the contamination characteristics of road networks, rivers and farmland within a certain range, determining the sources of contamination, effectively distinguishing their contamination levels and ecological risks, and providing land use and management bases for landowners.

Through the investigation of heavy metals around mining waste areas, farmland and ditches, the purposes of this study include to demonstrate the pollution status and ecological risk of each functional area. The objectives were: (1) to reveal the distribution and source of heavy metals in surrounding soils after long-term mining activities; (2) to assess the potential health and ecological risks of soil heavy metals; (3) to discuss the effects of topography, hydrology and land management on heavy metal pollution levels in soils.

## 2. Materials and Methods

### 2.1. Study Area

The investigation site is located in a Pb-Zn mine waste area, 5.5 km west of Lanping county town, in the longitudinal valley of the Hengduan Mountains in Northwestern Yunnan Province. The average altitude was 2880 m. The geographical coordinates were situated between 99°47′20″–99°47′97″ E, 26°45′19″–26°46′35″ N, with an average annual temperature of 10.7 °C and an average annual rainfall of 1002 mm. Wastes from mining activities over 100 years had caused heavy metal pollution to surrounding soils and watersheds.

### 2.2. Sample Collection

**Figure 1:**
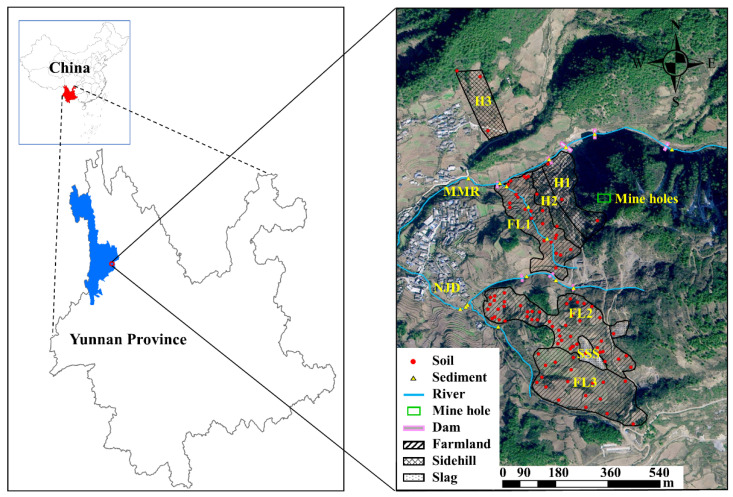
Location of the sampling sites.

114 sampling sites (Figure 1) in 9 functional areas (3 farmlands, 3 hillsides, 2 ditches and 1 slag stacking site) were surveyed in 2019 and the altitude of the sampling sites was recorded. The survey area was 0.5 km^2^ and the altitude ranged from 2800–2964 m. FL1, FL2 and FL3 were farmland 230 m west, 430 m south and 620 m south of the mine cave, respectively. H1, H2 and H3 were hillsides far from 130 m west, 200 m west and 350 m northwest of the mine cave, respectively. The slag stacking site (SSS) was located 500 m south of the mine cave. The Momian River (MMR) mainstream was located 175 m north of the cave and the tributary was located 300 m west of the cave. The Nanji Ditch (NJD) mainstream and tributary were located 315 m and 630 m southwest of the cave, respectively.

Soil/sediment samples consisted of five samples, which were collected from 0 to 20 cm depth in a 5 m × 5 m plot with the five-point method. Samples were well mixed and reduced to 1 kg after coning and quartering. 500 mL surface water was collected at the sediment sampling site with polyethylene plastic bottles before the sediment collected. Surface water was stored in 4 °C and determined in 1 day. The soil samples were filtered through 2 mm and 0.149 mm sieves after air-drying and stored in a glass desiccator protected from light in preparation for chemical analysis.

### 2.3. Chemical Analysis

Some 10.0 g soil samples were mixed with 25 mL pure water. Soil pH was determined with the supernatant of the mixture using a pH meter (Starter3100, OHAUS, Shanghai, China). Water pH was determined with a pH meter after mixing (Starter3100, OHAUS, Shanghai, China).

Some 1.0000 g soil samples were oxidized with K_2_Cr_2_O_7_ (1 M)-H_2_SO_4_ (95%) under heated conditions. Three drops of o-phenanthroline were added to digestion liquid and titrated with FeSO_4_ (0.5 M). The volume of FeSO_4_ consumed during the solution turned from orange to brick red was recorded. The content of organic matter (OM) in samples was calculated along with the amount of FeSO_4_ consumed. The calculation equation is as follows:

(1) $$OM\;\left({g\;\mathrm{kg}}^{-1}\right)=c\times \left(V_{0}-V\right)\times 10^{-3}\times 3.0\times 1.33\times 1.724/m\times 1000$$

where *OM* is the content of organic matter in the soil (g kg^−1^); *c* is the concentration of FeSO_4_; *V*_0_ is the volume of FeSO_4_ consumed by the blank sample (mL); *V* is the volume of FeSO_4_ consumed by the soil sample (mL); *m* is the weight of the soil sample (g).

Some 5.00 g of soil samples and 25.00 mL DTPA (0.005 M) were put into a 150 mL flask and oscillated for 2 h at 25 °C ± 2 °C and 180 r min^−1^ ± 20 r min^−1^. The filtrate was collected and the DTPA-extractable heavy metal concentrations determined by atomic absorption spectrometer (ICE 3300, Thermo Fisher, Bremerhaven, Germany).

Some 0.500 g of soil samples and 10 mL aqua regia (HNO_3_:HCl = 1:3) were put into a 150 mL flask. It was heated at a temperature of 140–160 °C until the brown smoke disappeared, 5 mL perchloric acid were added and heated to gray-white. The digestion liquid was collected and the heavy metal contents determined by atomic absorption spectrometer (ICE 3300, Thermo Fisher, Bremerhaven, Germany). Guaranteed reagents were used in the experiment. Standard reference soil (GBW07404, Cd 0.35 ± 0.08, 40 ± 4, Pb 58 ± 7, Zn 210 ± 19 mg kg^−1^) was used as a quality control. Recovery percentages were 92–110% for Cd, Cu, Pb and Zn. The analytical limits of Cd, Cu, Pb and Zn detection were 5, 4, 20 and 2 µg L^−1^, respectively. The gas flow rate of the ASS was set to 1.2 L min^−1^ with a burner height of 7 mm and an atomizer lift time of 4 s. The wavelength for Cd was 228.8 nm, Cu 324.8 nm, Pb 283.3 nm and Zn 213.9 nm.

A 100 mL well-mixed water sample was filtered through a 0.45 μm aqueous microporous filter membrane and stored, and the heavy metal content in the filtrate was the dissolved content in surface water. The surface water and filtrate were put into a 250 mL flask, with 5 mL nitric acid and 2 mL perchloric acid added, and heated to 1 mL on a hotplate. After cooling to room temperature, it was filtered into a 50 mL volumetric flask. The blank test was carried out at the same time. The heavy metals contents was determined by atomic absorption spectrometer (ICE 3300, Thermo Fisher, Bremerhaven, Germany). Guaranteed reagents were used in the experiment. Standard reference water (GSB07-1185-2000 for Cd 12.8 µg L^−1^, GSB07-1182-2000 for Cu 0.802 mg L^−1^, GSB07-1183-2000 for Pb 42.0 µg L^−1^, GSB07-1184-2000 for Zn 0.988 mg L^−1^) were used as quality control. Recovery percentages were 85–113% for Cd, Cu, Pb and Zn. The analytical limits of GFAAS method for Cd, Cu, Pb and Zn detection were 0.1, 0.4, 0.5 and 0.5 µg L^−1^, respectively. Argon gas flow rate was set to 0.2 L min^−1^. The sample detection process was 30 s in 110 °C for drying, 20 s in 400 °C for ashing, 3 s in 1300 °C for atomizing and 3 s in 2500 °C for residue removing. The wavelength for Cd was 228.8 nm, Cu 324.8 nm, Pb 283.3 nm and Zn 213.9 nm. The content of particulate heavy metals in the surface water was the content in the original solution excluding the content of heavy metals in the filtrate.

### 2.4. Semivariance Analysis

Semi-variogram analysis was used to describe the spatial characteristics of the coexistence of structural and stochastic characteristics of regionalized variables and to assess the spatial variability and correlation. GS+ was used to estimate the spatial variability of heavy metals in soil and sediment.

(2) $$\gamma \left(h\right)=\left[1/2N\left(h\right)\right]\Sigma_{i=1}^{N\left(h\right)}Z\left(x_{i}+h\right)-Z\left(x_{i}\right)]h^{2}$$

where, =*γ*(*h*) is the experimental semi-variance value for all pairs at a lag distance *h*; *Z* (*x_i_*) is the soil heavy metal content at point *i*; *Z* (*x_i_ + h*) is the soil heavy metal content at point *i + h*. The value of the semi-variogram is the mean of the squares of the difference between the attributes of sample point *x* and sample point *h*. 

The calculated semi-variance function values can be fitted by a series of theoretical models and characterized by nugget variance (C0), Sill (C0+C) and range, which represent the measurement error or spatial variation, the maximum variance between data pairs and the farthest distance of correlation between graphic parameters, respectively. The nugget to sill (N:S ) ratio [C0/(C0+C)] of ≤0.25 means strong spatial dependency, which indicate the variation of heavy metals mainly affected by the structural effect of the natural environment; the ratio remains between 0.25 and 0.75 which means moderate spatial dependency, indicating the variation of heavy metals mainly affected by the joint action of the natural environment factors and the random factors of human activities; and the ratio of ≥0.75 suggests weak spatial dependency, which indicates the variation of heavy metals mainly affected by human activities [20,21]. Main natural factors include climate, parent material, topography and soil properties, and human activities including fertilization, farming measures and cropping systems.

### 2.5. Pollution Assessment

Nemerow index and HAKANSON method [22] were used to evaluate soil heavy metal pollution and potential ecological risk of the survey sites. Calculated as follows:

(3) $$P_{i}=C_{i}/B_{i}$$

where *P_i_* is single pollution index of a particular heavy metal; *C_i_* is content of a particular heavy metal; *B_i_* is the background value of heavy metals in soil in Yunnan (Cd 0.22, Pb 40.60, Cu 46.30, Zn 89.70 mg kg^−1^) [23]. Pi is classified to class 0 (*P_i_* < 1) non-contamination, 1 (1 ≤ *P_i_* < 2) slight contamination, 2 (2 ≤ *P_i_* < 3) low contamination, 3 (3 ≤ *P_i_* < 5) moderate contamination and 4 (*P_i_* ≥ 5) heavy contamination.

(4) $$I_{N}=\sqrt{\left[{\left(\bar{P_{i}}\right)}^{2}+{\left(P_{imax}\right)}^{2}\right]/2}$$

where *I_N_* is Nemerow index; *P_imax_* is the maximum *P_i_* value of all metals in a sample; and $\bar{P_{i}}$ is the arithmetic mean of the *P_i_*. *I_N_* is classified to class 0 (*I_N_* < 0.7) non-contamination, 1 (0.7≤ *I_N_* < 1) slight contamination, 2 (1 ≤ *I_N_* < 2) low contamination, 3 (2 ≤ *I_N_* < 3) moderate contamination and 4 (*I_N_* ≥ 3) heavy contamination.

(5) $$I_{geo}=log_{2}\left(C_{i}/1.5B_{i}\right)$$

where *I_geo_* is the geo-accumulation index [24]; *C_i_* is the measured concentration of heavy metal in samples; *B_i_* is the background value of heavy metals in soil in Yunnan; Factor 1.5 was used to correct possible changes in background values for specific metals in the environment. *I_geo_* is classified to class 0 (*I_geo_* < 0) non-contamination, 1 (0 < *I_geo_* < 1) slight contamination, 2 (1 ≤ *I_geo_* < 2) low contamination, 3 (2 ≤ *I_geo_* < 3) moderate contamination, 4 (3 ≤ *I_geo_* < 4) heavy contamination, 5 (4 ≤ *Igeo* < 5) high contamination, 6 (*I_geo_* ≥ 5) extreme contamination.

(6) $$I_{IN}=\sqrt{{\left(I_{geomean}\right)}^{2}+{\left(I_{geomax}\right)}^{2}}\;$$

where *I_IN_* is the improved Nemerow index [25]; *I_geomax_* is the maximum *I_geo_* value of all metals in a sample; *I_geomean_* is the mean of the *I_geo_*. *I_IN_* is classified to class 0 (*I_IN_* < 0.5), 1 (0.5 ≤ *I_IN_* < 1), 2 (1 ≤ *I_IN_* < 2), 3 (2 ≤ *I_IN_* < 3), 4 (3 ≤ *I_IN_* < 4), 5 (4 ≤ *I_IN_* < 5) and 6 (*I_IN_* ≥ 5).

(7) $$mCd=\Sigma_{i=1}^{n}\left(Ci/Bi\right)/n$$

where *mCd* is the modified degree of contamination [26]; *C_i_* is the measured concentration of heavy metal in samples; *B_i_* is the background value of heavy metal in soil. *mCd* is classified as very low (*mCd* < 1.5), low (1.5 ≤ *mCd* < 2), moderate (2 ≤ *mCd* < 4), high (4 ≤ *mCd* < 8), very high (8 ≤ *mCd* < 16), extremely high (16 ≤ *mCd* < 32), and ultra-high (*mCd* ≥ 32).

(8) $$PLI=\sqrt[{n}]{\Pi_{i=1}^{n}C_{i}/B_{i}}$$

where *PLI* is the pollution load index [27]; *C_i_* is the measured concentration of heavy metal in samples; *B_i_* is the background value of heavy metal in soil. *PLI* is classified as class low (*PLI* < 1), moderate (1 ≤ *PLI* < 2), high (2 ≤ *PLI* < 5) and very high (*PLI* ≥ 5).

(9) $$E_{r}^{i}=T_{r}^{i}\times C_{f}^{i}\;$$

where $E_{r}^{i}$ is the potential ecological risk hazard index; $T_{r}^{i}$ is the toxicity response coefficient of heavy metals (Cd = 30, Cu = Pb = 5, Zn = 1); $C_{f}^{i}$ is the contamination factor ($C_{f}^{i}$ = *C_i_*/*B_i_*); *C_i_* is the measured concentration of heavy metal in samples; *B_i_* is the background value of heavy metal in soil. $E_{r}^{i}$ is classified as low risk of contamination ($E_{r}^{i}$ < 40), moderate risk of contamination (40 ≤ $E_{r}^{i}$ < 80), considerable risk of contamination (80 ≤ $E_{r}^{i}$ < 160), high risk of contamination (160 ≤ $E_{r}^{i}$ < 320) and extreme risk of contamination ($E_{r}^{i}$ ≥ 320).

(10) $$RI=\Sigma_{i=1}^{n}E_{r}^{i}\;$$

where *RI* is comprehensive ecological risk index. *RI* is classified as low potential ecological risk (*RI* < 150), moderate potential ecological risk (150 ≤ *RI* < 300), considerable potential ecological risk (300 ≤ *RI* < 600) and extreme potential ecological risk (*RI* ≥ 600).

### 2.6. Exposure Assessment

The hazard quotient (*HQ*) and hazard index (*HI*) recommended by the United States Environmental Protection Agency (USEPA) were used to assess the health risk to children and adults. Direct ingestion, dermal contact and inhalation absorption were the main pathways of human exposure associated with soil heavy metals. The average daily human exposure *ADI_ing_*, *ADI_dermal_* and *ADI_inh_* (mg kg^−1^ d^−1^) [28] was calculated as follows:

(11) $$ADI_{ing}=\frac{C_{s}\times IngR\times EF\times ED}{BW\times AT}\times 10^{-6}\;$$

(12) $$ADI_{dermal}=\frac{C_{s}\times SA\times AF\times ABS\times EF\times ED}{BW\times AT}\times 10^{-6}\;$$

(13) $$ADI_{inh}=\frac{C_{s}\times InhR\times EF\times ED}{BW\times PEF\times AT}\;$$

where *C_s_* is the measured concentration of heavy metal in samples (mg kg^−1^); *IngR* is the ingestion rate of soil (mg day^−1^); *EF* is the exposure frequency (d year^−1^); *ED* is exposure duration (year); *BW* is the average weight of the exposed individual (kg); *AT* is the average exposure time (d); *SA* is the exposed skin surface area (cm^2^); *AF* is the skin adherence factor (mg cm^−2^); *ABS* is the dermal absorption factor (unitless); *InhR* is the inhalation rate (m^3^ day^−1^); *PEF* is the emission factor (m^3^ kg^−1^). The value of each parameter refers to the USEPA exposure factor manual.

Non-cancer risk and cancer risk due to heavy metal element were calculated by hazard indices *HQ* and *CR* and non-cancer risk and cancer risk due to multiple heavy metal elements were calculated by combined hazard indices HI and CRI [29].

(14) $$HI=\Sigma HQ_{i}=\Sigma (ADI_{i}/RFD_{i})\;$$

(15) $$CRI=\Sigma CR_{i}=\Sigma (ADI_{i}\times SF_{i})$$

where *ADI_i_* is the average daily exposure of *i* heavy metal under exposure routes (m^3^ kg^−1^ d^−1^); *RFD_i_* is the reference dose of each metal under *i* exposure routes (m^3^ kg^−1^ d^−1^); *SF_i_* is the slope factor of carcinogenic risk under *i* exposure routes (mg kg^−1^ d^−1^). If *HQ* < 1 and *HI* < 1, the non-carcinogenic health risk is negligible. *CR* and *CRI* surpassing 1 × 10^−4^ means unacceptable carcinogenic risk, below 1 × 10^−6^ means no carcinogenic risk and lying between 1 × 10^−4^ and 1 × 10^−6^ means carcinogenic risk for heavy metal content in soil is within an acceptable range.

### 2.7. Data Statistical Analysis

Excel 2016 was used for data analysis including charts and descriptive statistics. Origin 8.0 was used for plotting. GIS software was used to construct spatial distribution maps of the heavy metal concentrations. ANOVA, correlation and regression analysis between heavy metals were used in SPSS 19 statistical software and the significant threshold was set at *p* < 0.05 (significant) and *p* < 0.01 (extremely significant). Spatial distribution maps of heavy metals were constructed with GIS software.

## 3. Results

### 3.1. pH and OM Contents

Soil pH and organic matter contents of the samples are shown in Table 1. Soil pH ranged from 5.45–9.66 with a mean value of 7.21. Samples of pH < 6.5, 6.5 ≤ pH ≤ 7.5 and 7.5 < pH accounted for 22%, 33% and 45% of the total samples, respectively, whereas 100% of the acidic samples and 76% of the neutral samples were in FL2 and FL3 and the pH of the samples in these two areas was significantly lower than those of other areas (*p* < 0.05). OM contents ranged from 0.97–78.71 g kg^−1^ with a mean value of 33.52 g kg^−1^. OM contents in farmland areas were significantly higher than those in H1, H3, NJD and SSS (*p* < 0.05).

### 3.2. Heavy Metal Contents

**Figure 2:**
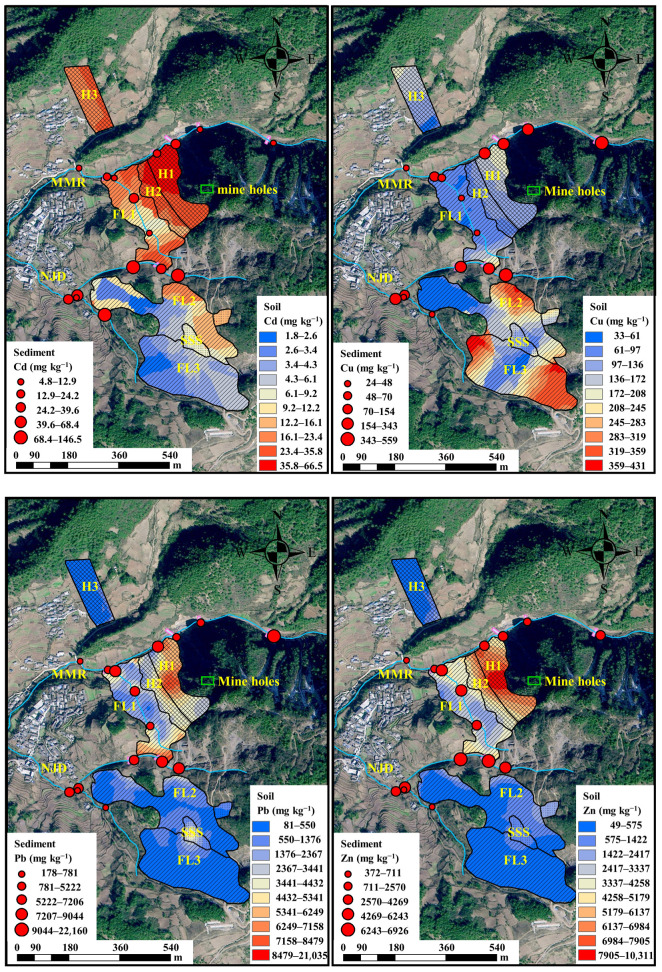
Heavy metal content of samples in different regions.

Heavy metal contents showed spatial and elemental specificity (Figure 2). The mean contents of Cd, Cu, Pb and Zn were 15.35, 154.0, 1919 and 1667 mg kg^−1^, respectively, with coefficients of variation 133–192%. The average contents of total heavy metals were 69.75, 3.33, 47.26 and 18.58 times of the geochemical background value (Cd 0.22, Pb 40.60, Cu 46.30, Zn 89.70 mg kg^−1^) found in the soil of Yunnan, respectively [23]. 100% samples for Cd, 61% for Cu, 100% for Pb and 97% Zn samples exceeded the Yunnan geochemical background values of the heavy metals in the soil. The mean contents of Cd and Zn of the samples in H1 were 127.1 mg kg^−1^ and 8699 mg kg^−1^ and significantly higher than those in other areas (*p* < 0.05). Cu content of the samples in FL3 was 295.4 mg kg^−1^. Pb content of the samples in MMR was 7341 mg kg^−1^, and significantly higher than those in other areas, except for NJD (*p* < 0.05). The average contents of Cd, Cu, Pb and Zn in H1, H1, FL3 and MMR were 557.6, 96.37, 6.37 and 180.8 times of the Yunnan geochemical background values (Cd 0.22, Pb 40.60, Cu 46.30, Zn 89.70 mg kg^−1^). The mainstream of MMR was close to H1. After 5 dam interceptions, the contents of Cu, Pb and Zn in the downstream sediment decreased by 45%, 78% and 70% compared with those in the upstream, respectively. Cu, Pb and Zn in sediments at the tributary of the MMR presented accumulation at the front of the estuary, and Cu presented cumulative effect due to the presence of artificial dams at the estuary 385 m from the mine. The mainstream of NJD was close to H2, FL1 and FL2, and the contents of heavy metals in sediments largely ranged at the corner (645–675 m from the mine holes) of the NJD.

DTPA-extractable contents of heavy metals differed significantly depending on land management practices and geographical location (Table 2). The mean DTPA-extractable Cd, Cu, Pb and Zn contents were 1.56, 14.03, 249.7 and 99.16 mg kg^−1^ with percentages of 25%, 15%, 27% and 7% of total contents. The percentage of DTPA-extractable for Cd in FL1and FL2, for Pb in SSS, FL2 and FL3 was more than 30%.

### 3.3. Heavy Metal Contents in Surface Water

The heavy metal contents and pH of surface water in the ditches were analyzed (Table 3). The contents of Cd, Cu, Pb and Zn in the ditches were 0.16–1.35, 3.14–12.69, 1.85–14.77 and 156.8–1366 μg L^−1^, respectively, and the dissolved contents accounted for 70%, 53%, 23% and 58% of the total contents. Heavy metal contents of surface water in the ditches were all below the standard limits of surface water environmental quality standard V of China [30]. Dissolved Cd content, total Cd contents and pH in the surface water of NJD were significantly higher than those of MMR (*p* < 0.05).

### 3.4. Relationships between Soil Chemical Properties, Altitude and Distance

Soil OM content had an extremely significant negative correlation with pH, Cd, Pb and Zn contents (*p* < 0.01) (Table 4). This means the contents of Cd, Pb and Zn were low in fertile areas (FL1, FL2, FL3 and H3). Negative correlations between elevation and content of Pb, Zn, and DTPA-extractable contents of Pb and Zn (*p* < 0.05) were observed. There was extremely significant positive correlation between elevation and Cu contents (*p* < 0.01).

**Figure 3:**
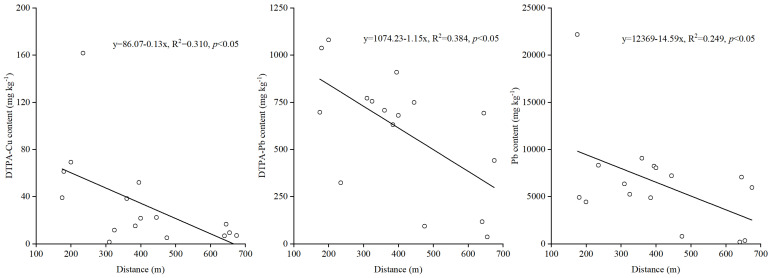
Linear regression analysis between heavy metal contents in sediment and the distance from the sampling sites to the mine holes.

DTPA-extractable contents of Cu and Pb in sediment and total Pb contents in sediment had a negative relationship with the distance from the sampling sites to the mine holes (*p* < 0.05) (Figure 3).

### 3.5. Semi-Variogram Analysis of Heavy Metals in Soil and Sediment

**Figure 4:**
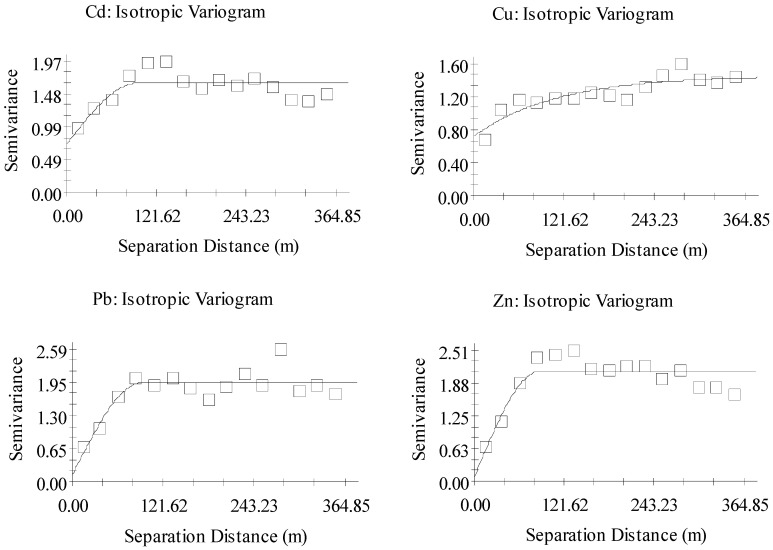
Semi-variance modeling of four kinds of total heavy metals.

Semi-variogram analysis was used to assess the spatial variability and correlation. The optimal semi-variogram model was selected based on the principles of maximum R^2^, minimum RSS and range greater than the sample spacing, and the relevant parameters were obtained (Table 5, Figure 4). Cd, Cu, Pb and Zn were log-transformed using GS+ and the transformed data were consistent with the assumptions of geostatistical analysis. Cu conformed to the exponential model and Cd, Pb and Zn to the spherical model. Zn (0.043) and Pb (0.068) showed strong spatial dependency (N:S < 0.25), indicating that the spatial distribution of Zn and Pb in mine wastes area were mainly influenced by structural factors. Cd (0.436) and Cu (0.494) showed moderate spatial dependency (0.25 < N:S < 0.75), caused by both structural and random factors. The range value of inconsistent distribution pattern varied from 89 m to 96 m for Cd, Pb and Zn, and 338 m for Cu.

### 3.6. Assessment of Environmental Risks

Integrated pollution assessment indicated that topography had a significant effect on the degree of contamination. Areas near mine holes showed a high degree of pollution. Ditches received pollutants from mine waste areas and acted as barriers to slow pollutant spread to opposite riverbanks. H1, MMR and NJD were the most polluted areas. Cd was the most serious pollution in mine wastes areas, followed by Pb, Zn and Cu (Table 6).

The pollution level of the mine waste areas was evaluated using the pollution factor and Nemerow Index and presented in Table 6. The results showed that all samples were contaminated with Cd and Pb. The maximum *P_i_* values of Cd, Cu, Pb and Zn were found in H1 (797.6), FL3 (35.36), MMR (545.8) and H1 (114.7), and 95.65%, 25.00%, 80.43% and 42.39% of the samples were heavily contaminated, respectively. All sampling sites were heavily contaminated with Cd and with Pb except for H3. FL3 was heavily contaminated with Cu. FL2, FL3 and H3, away from the mine cave, were not heavily contaminated with Zn. Some 22.83% of the samples showed extreme contamination, mainly in H1, H2, MMR, NJD and SSS. Sampling was mainly on the slopes, mining waste area and ditches. Mining activities caused the accumulation of heavy metals in these areas and the farmlands further away from the mine holes were less affected.

Values of *I_N_*, *PLI*, *mCd* and *I_IN_* for all samples ranged from 2.55–598.28, 1.71–283.37, 1.13–104.19 and 0.72–7.71. All sampling areas are extremely polluted. H1, H2, MMR, NJD, SSS are extremely polluted based on *PLI*, *mCd* and *I_IN_*. The contaminants were mainly Cd and Pb.

**Figure 5:**
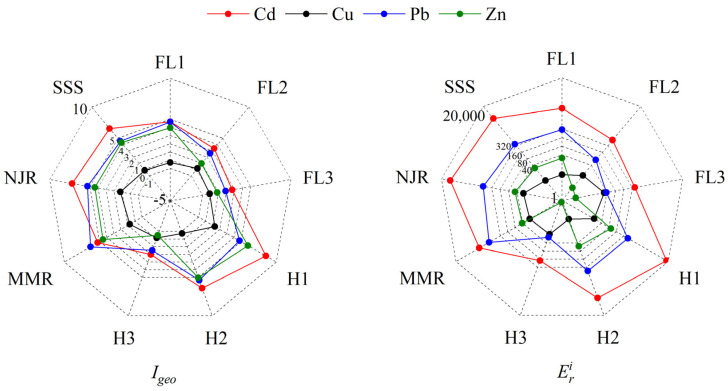
*I_geo_* and $E_{r}^{i}$ of heavy metals.

The geo-accumulation index (Figure 5) in areas near the mine cave and the ditch *I_geo_* were high. *I_geo_* ranged from −3.08 to 9.05 with mean values of Cd (4.00) > Pb (3.18) > Zn (1.87) > Cu (0.25). Cd, Cu, Pb and Zn accounted for 0%, 52.17%, 0% and 10.87% of non-contaminated, 27.17%, 41.30%, 58.70% and 65.22% of slightly to moderately contaminated and 72.83%, 6.52%, 41.30% and 23.91% of more than heavily contaminated, respectively. H1 and H2 were extremely contaminated for Cd, Pb and Zn, MMR and NJD for Cd and Pb, and SSS for Cd. H3 has the lowest *I_geo_* values and the lowest contamination levels for Cd, Cu, Pb and Zn.

### 3.7. Potential Ecological Risk Index

The results of the ecological risk assessment of heavy metals in soil and substrates by $E_{r}^{i}$ showed that 78.07% of the samples had extreme risk from Cd and 24.56% had high risk from Pb (Figure 5). Cu and Zn were a low contamination risk with 89.47% and 83.33%, and no extreme risk samples. H3 was high risk for Cd and other areas were at extreme risk of contamination for Cd. All areas were low contamination risk from Cu. FL3 and H3 were low risk of Pb contamination, FL2 was at medium risk of Pb contamination, FL1 was at high risk of Pb contamination, and all other areas were at extreme risk of Pb contamination. H1 was at high risk of Zn contamination, H2, MMR and NJD were at medium risk of Zn contamination and all other areas were at low risk of Zn contamination. Cd and Pb contributed to extreme risk of contamination. All areas at very high risk of contamination were adjacent to mine caves.

*RI* was an important indicator to assess the potential ecological risk by comprehensively involving the content and toxicity of the target heavy metals. The percentage of samples with low potential ecological risk, medium potential ecological risk, high potential ecological risk and very high potential ecological risk were 3.51%, 11.40%, 28.95% and 56.14%, respectively. The ranking of the combined ecological risk index in the survey area was H1 > NJR > SSS > H2 > MMR > FL1 > FL2 > FL3 > H3. H3 was a moderate potential ecological risk, FL3 was considerable potential ecological risk, and other areas were extreme potential ecological risk. Ecological risk from Cd was the main factor constituting ecological risk in the survey area, and the contribution of heavy metals to *RI* was Cd > Pb > Zn > Cu in order. The ecological risk posed by these heavy metals spread to low terrain with a tendency to decrease with distance. Pollutants migrating from mine holes and mine wastes area to ditches constituted an ecological risk in the watershed and spread downstream. These risks were reduced due to distance, topography and artificial dams.

### 3.8. Human Health Risk Assessment of Heavy Metal Pollution

The health risks caused by heavy metals in soil and sediment were calculated by exposure index (*ADD*), non-carcinogenic risk index (*HQ*) and carcinogenicity index (*CR*) (Table 7). The basic trend of mean *HQ* values was Pb > Cd > Zn > Cu. There was a non-carcinogenic risk for children with a *HI* value of 3.69. *HQ* for direct ingestion of Pb was 3.63. The *HI* values for adults were less than 1, indicating that non-carcinogenic risk for adults was negligible. The mean *CR* values of Cd and Pb were 4.48 × 10^−5^ and 7.85 × 10^−6^ for adults and 4.86 × 10^−^^5^ and 8.50 × 10^−6^ for children. Direct ingestion was an important route of exposure to heavy metals.

## 4. Discussion

Land management measures have led to strong variation in pH values and OM contents of soil/sediment in different functional areas. Soil fertility was improved with the long-term application of monocalcium phosphate, potassium sulphate and farmyard manure, and pH was decreased in farmland. This may have led to an increase in bioavailable heavy metals, whose transfer to crops was continuous [31].

The destruction of the surface vegetation led to an increase in the probability of waste area erosion and the diffusion of mining wastes resulted in higher Cd, Pb and Zn contents in the surface soil and ditch sediment. Terrain (elevation, slope and ditch) was the main reason for the low content of H3 heavy metals, which created geographical isolation [32]. Semi-variogram analysis indicated that the spatial distribution of Cd and Cu in the surveyed area was caused by parent material, topography, soil properties and mining, with the ratio of N:S between 0.25–0.75; Pb and Zn were mainly influenced by mining with the ratio of N:S lower than 0.25 [33]. Away from the mine cave, agricultural practices and clean soil applications buffered the damage of heavy metals to farmland [34]. Soil amelioration and remediation based on the bio-geosystem technique (BGT*) was a potential remediation step [35].

H1 and H2 were close to the mine cave and mining waste covered the surface areas. The DTPA-extractable contents of Cd and Pb were high in H1 and H2 leading to a migration from hillslopes to ditches. A similar result in the dry-hot valley of Upper Red River in southwestern China was obtained, in that the contents of Zn and Pb are higher in low altitude areas and the spatial distribution pattern of Cu is opposite to this [36]. Cu and Zn were less mobile, but also accumulated to some extent in the ditches.

Heavy metal migration showed heterogeneity. There was no rainfall during the survey period and 70% Cd, 58% Zn, 53% Cu, and 23% Pb carried by surface water were dissolved. Cd migrated mainly in the dissolved form, Cu and Zn in the dissolved and particulate forms and Pb in the particulate form in surface water on non-rainy days. It was found that large amounts of heavy metals mainly migrated in particulate form in surface water during rainfall [37]. Ditches and slopes were not conducive to heavy metal control due to elevation differences. Cd and Pb were selected as the priority pollutants to be controlled. Measures should be taken to block the accumulation path of pollutants from the slope to the ditch, weakening the ability of water flow shock to transport heavy metals in ditches.

MMR and NJD accommodated Cd and Pb migrating from the slope and mining waste area. Artificial measures, terrain, the distance from the sampling sites to the mine holes and topography influenced the variability in the dispersal behavior and the migration processes of heavy metals in the ditch. Flow sorting resulted in smaller sediment particle size in high-velocity and low-lying channels and the accumulation of silt and clay was obvious at the corners (655 m away from the mine holes) in NJD [38]. Heavy metals accumulated in clay and migrated with the scour of water flow, which caused low heavy metal contents at the corner of ditches and a large variation of heavy metal contents 645–675 m from the mine holes [39].

The mean contents of Cd, Cu, Pb and Zn were 3.72, 11.29, 4.84 and 2.93 times the recommended geochemical baseline values of Lanping [25], respectively. Based on the geo-accumulation index, the highest *I_geo_* values were in ditches and side slopes. Cd and Pb were recommended as priority heavy metals considering remediation. The ecological risk assessment showed that sediment in ditches was an important risk source. Management measures should be taken to avoid the spread of risk sources. Recommended measures include increasing vegetation cover of the slope, building dams and removing sedimentation [40,41,42].

Carcinogenic risk of Cd for adults and children cannot be negligible. The non-cancer risk of Pb for children exceeded the USEPA recommendation value. Cd and Pb presented potential health risks to natives in the surveyed area and should be of particular concern. Direct ingestion (mainly food intake) was the major source of health risk. Growing low accumulation maize and beans was a suitable agricultural measure. The most effective ways to protect children from heavy metals included restraining children’s behavior, avoiding pica, finger or hand sucking, reducing oral ingestion of heavy metals in soil that pose health risks to children and staying away from contaminated areas [43]. Ditches were the main areas posing health risks and human activity in ditches should be avoided.

## 5. Conclusions

In conclusion, the distribution characteristics, sources and health risks of heavy metals were researched in the Lanping Pb-Zn mining abandoned area. Results revealed that Cd and Cu showed moderate spatial dependency. Pb and Zn showed strong spatial dependency. Soil and sediment were polluted with Cd, Cu, Pb and Zn to different degrees. The *I_geo_* of heavy metals were Cd (4.00) > Pb (3.18) > Zn (1.87) > Cu (0.25). There was extreme potential ecological risk in the investigation area, with Cd being the main factor causing extremely high risk, followed by Pb, Zn and Cu. Non-carcinogenic risk to children from Pb and carcinogenic risk to adult and children from Cd was non-negligible. Sediment in the watershed posed a wider radiation range than in agricultural land and slopes. The health risks to natives caused by the migration of heavy metals through ditches were via diffusion. It is recommended to reduce the spread of pollutants and ecological risks through ecological restoration and ecological buffer zone construction.

## Figures and Tables

**Table 1:** pH and OM contents in different regions.

Investigated Area	pH		OM(g kg^−1^)	
	Mean ± SD	CV	Mean ± SD	CV
FL1	8.19±0.73 a	8.97%	36.09±19.88 ab	55.08%
FL2	6.76±0.65 b	9.65%	41.91±13.05 a	31.14%
FL3	6.79±1.13 b	16.65%	36.81±11.66 ab	31.68%
H1	7.71±0.25 ab	3.26%	13.70±10.10 c	73.69%
H2	8.77±0.97 a	11.03%	39.23±24.75 a	63.09%
H3	8.18±1.20 a	14.64%	11.50±5.42 c	47.18%
MMR	8.70±0.99 a	11.41%	18.92±8.19 bc	43.30%
NJD	7.93±0.50 a	6.34%	9.13±2.87 c	31.42%
SSS	7.83±0.04 ab	0.45%	12.27±15.98 c	130.20%

Note: The different lowercase letters indicate significant difference of the pH and OM content between different areas at *p* < 0.05 level.

**Table 2:** DTPA-extractable heavy metal contents and percentage of the total contents (mg kg^−1^).

Investigated Area	Cd		Cu		Pb		Zn	
	Content	Percentage	Content	Percentage	Content	Percentage	Content	Percentage
FL1	2.16 ± 2.00 b	1–86%	8.93 ± 7.06 b	2–30%	496.9 ± 401.1 ab	1–51%	254.4 ± 222.0 ab	0–24%
FL2	1.32 ± 1.05 b	1–76%	10.57 ± 10.09 b	1–68%	162.8 ± 143.6 c	7–72%	25.18 ± 38.93 cd	0–25%
FL3	0.44 ± 0.33 b	3–62%	9.17 ± 10.99 b	0–53%	82.37 ± 59.77 c	9–72%	26.48 ± 23.03 cd	0–29%
H1	6.14 ± 3.38 a	3–6%	10.75 ± 5.99 b	3–11%	260.2 ± 125.8 bc	2–56%	456.6 ± 195.5 a	3–11%
H2	2.23 ± 0.24 b	4–15%	9.00 ± 4.07 b	12–40%	672.4 ± 335.7 a	13–29%	361.5 ± 363.4 ab	1–10%
H3	0.24 ± 0.27 b	2–33%	7.53 ± 3.78 b	2–24%	32.79 ± 15.43 c	8–22%	6.05 ± 4.83 d	1–23%
MMR	1.62 ± 0.67 c	3–35%	44.71 ± 50.04 a	4–62%	676.8 ± 311.5 a	3–24%	345.8 ± 300.2 ab	0–23%
NJD	4.24 ± 3.40 a	0–13%	19.35 ± 15.79 b	3–25%	517.5 ± 332.2 ab	7–66%	227.5 ± 275.4 bc	0–17%
SSS	1.64 ± 0.22 b	3–10%	4.48 ± 2.29 b	5–8%	615.8 ± 591.4 a	18–45%	143.1 ± 80.8 bc	3–8%

Note: Different lowercase letters indicate significant differences of the EDTA-extractable heavy metal contents between different areas at *p* < 0.05 level.

**Table 3:** Heavy metal contents of surface water in ditches (μg L^−1^).

Investigated Sites	Dissolved				Total				
	Cd	Cu	Pb	Zn	Cd	Cu	Pb	Zn	pH
MMR	0.31 ± 0.19 b	2.98 ± 1.56 a	1.35 ± 0.96 a	321.9 ± 473.4 a	0.47 ± 0.25 b	5.85 ± 3.53 a	5.06 ± 3.05 a	406.0 ± 475.7 a	8.33 ± 0.05 b
NJD	0.68 ± 0.31 a	4.42 ± 2.29 a	1.57 ± 0.73 a	245.5 ± 169.3 a	0.99 ± 0.24 a	8.28 ± 2.86 a	9.07 ± 3.46 a	421.3 ± 187.9 a	8.50 ± 0.11 a

Note: Different lowercase letters indicate significant differences of the heavy metal contents between different ditches at *p* < 0.05 level.

**Table 4:** Correlation analysis between altitude, pH, heavy metal content and OM content of samples.

	Altitude	pH	OM	DTPA-Cd	DTPA-Cu	DTPA-Pb	DTPA-Zn	Cd	Cu	Pb	Zn
Altitude	1.000										
pH	−0.316 **	1.000									
OM	−0.001	−0.360 **	1.000								
DTPA-Cd	−0.179	0.294 **	−0.165	1.000							
DTPA-Cu	0.004	0.248 **	−0.128	0.132	1.000						
DTPA-Pb	−0.199 *	0.416 **	−0.155	0.417 **	0.316 **	1.000					
DTPA-Zn	−0.261 **	0.437 **	−0.134	0.329 **	0.484 **	0.576 **	1.000				
Cd	−0.123	0.205 *	−0.351 **	0.642 **	0.138	0.280 **	0.494 **	1.000			
Cu	0.249 **	−0.064	−0.109	0.021	0.286 **	0.030	0.006	0.049	1.000		
Pb	−0.210 *	0.488 **	−0.271 **	0.398 **	0.415 **	0.638 **	0.529 **	0.400 **	0.147	1.000	
Zn	−0.319 **	0.515 **	−0.300 **	0.719 **	0.233 *	0.636 **	0.711 **	0.741 **	0.024	0.681 **	1.000

Note: “*” and “**” indicate significant correlation at *p* < 0.05 and *p* < 0.01 level according to Pearson correlation analysis, respectively.

**Table 5:** Semi-variogram parameters of optimal geostatistical models.

Parameters	Best-Fit Model	Nugget (C0)	Sill (C + C0)	Range (m)	N:S (C0/(C + C0))	Spatial Dependence	R^2^	RSS
LogCd	Spherical	0.715	1.64	96	0.436	Moderate	0.565	0.418
LogCu	Exponential	0.717	1.45	338	0.494	Moderate	0.772	0.149
LogPb	Spherical	0.132	1.93	94	0.068	Strong	0.746	0.703
LogZn	Spherical	0.091	2.10	89	0.043	Strong	0.766	0.783

**Table 6:** *Pi*, *I_N_*, *PLI*, *mCd* and *I_IN_* of heavy metals.

Investigated Area	*P_i_*				*I_N_*	*PLI*	*mCd*	*I_IN_*
	Cd	Cu	Pb	Zn				
FL1	57.83	1.61	60.46	30.49	61.04	18.17	37.60	4.37
FL2	19.33	2.75	14.20	3.75	17.24	6.49	10.01	2.82
FL3	13.10	6.38	7.62	3.12	12.92	4.93	7.55	2.37
H1	577.6	3.99	94.34	96.98	430.7	61.91	193.2	7.04
H2	150.7	1.02	88.70	52.43	118.5	27.34	73.21	5.34
H3	6.05	3.73	4.85	1.17	6.13	2.75	3.95	1.74
MMR	75.29	4.09	180.8	41.32	141.4	33.04	75.38	5.43
NJD	325.4	4.78	130.1	47.37	249.8	51.44	126.9	6.06
SSS	188.2	1.62	75.75	30.05	168.6	22.49	73.91	5.67

**Table 7:** Non-cancer and cancer risk value.

**Non-Carcinogenic Risk**	**Direct Ingestion**		**Dermal Contact**		**Inhalation Absorption**		***HI***	
	**Adults**	**Children**	**Adults**	**Children**	**Adults**	**Children**	**Adults**	**Children**
*HQ_Cd_*	2.34 × 10^−2^	1.01 × 10^−1^	9.32 × 10^−3^	2.84 × 10^−3^	3.43 × 10^−4^	1.18 × 10^−3^	3.30 × 10^−2^	1.06 × 10^−1^
*HQ_Cu_*	5.86 × 10^−3^	2.55 × 10^−2^	7.79 × 10^−5^	7.13 × 10^−5^	8.58 × 10^−7^	1.12 × 10^−4^	5.94 × 10^−3^	2.56 × 10^−2^
*HQ_Pb_*	8.34 × 10^−1^	3.63	2.22 × 10^−2^	6.77 × 10^−2^	1.22 × 10^−4^	1.01 × 10^−4^	8.57 × 10^−1^	3.69
*HQ_Zn_*	8.46 × 10^−3^	3.67 × 10^−2^	1.69 × 10^−4^	5.14 × 10^−4^	1.24 × 10^−6^	1.03 × 10^−6^	8.63 × 10^−3^	3.73 × 10^−2^
*HI_HMs_*	8.72 × 10^−1^	3.79	3.18 × 10^−2^	7.11 × 10^−2^	4.68 × 10^−4^	1.39 × 10^−3^	9.04 × 10^−1^	3.86
**Carcinogenic Risk**	**Direct Ingestion**		**Dermal Contact**		**Inhalation Absorption**		***CRI***	
	**Adults**	**Children**	**Adults**	**Children**	**Adults**	**Children**	**Adults**	**Children**
*CR_Cd_*	4.46 × 10^−5^	4.85 × 10^−5^	1.78 × 10^−7^	1.36 × 10^−7^	6.78 × 10^−9^	5.60 × 10^−9^	4.48 × 10^−5^	4.86 × 10^−5^
*CR_Pb_*	7.78 × 10^−6^	8.45 × 10^−6^	6.21 × 10^−8^	4.73 × 10^−8^	5.65 × 10^−9^	4.67 × 10^−9^	7.85 × 10^−6^	8.50 × 10^−6^
*CRI_HMs_*	5.24 × 10^−5^	5.69 × 10^−5^	2.40 × 10^−7^	1.83 × 10^−7^	1.24 × 10^−8^	1.03 × 10^−8^	5.27 × 10^−5^	5.71 × 10^−5^

## Data Availability

Not applicable.
